# Faecal Proteomics and Functional Analysis of Equine Melanocytic Neoplasm in Grey Horses

**DOI:** 10.3390/vetsci9020094

**Published:** 2022-02-21

**Authors:** Parichart Tesena, Amornthep Kingkaw, Narumon Phaonakrop, Sittiruk Roytrakul, Paviga Limudomporn, Wanwipa Vongsangnak, Attawit Kovitvadhi

**Affiliations:** 1 Graduate Student in Animal Health and Biomedical Science Program, Faculty of Veterinary Medicine, Kasetsart University, Bangkok 10900, Thailand; parichart.tes@mahidol.ac.th; 2 Department of Clinical Science and Public Health, Faculty of Veterinary Science, Mahidol University, Salaya, Puttamonthon, Nakhon Pathom 73170, Thailand; 3 Interdisciplinary Graduate Program in Bioscience, Faculty of Science, Kasetsart University, Bangkok 10900, Thailand; amornthep.ki@ku.th; 4 Functional Ingredients and Food Innovation Research Group, National Center for Genetic Engineering and Biotechnology, National Science and Technology Development Agency, Pathum Thani 12120, Thailand; narumon.pha@biotec.or.th (N.P.); sittiruk@biotec.or.th (S.R.); 5 Department of Zoology, Faculty of Science, Kasetsart University, Bangkok 10900, Thailand; fscipil@ku.ac.th; 6 Omics Center for Agriculture, Bioresources, Food, and Health, Kasetsart University (OmiKU), Bangkok 10900, Thailand; 7 Department of Physiology, Faculty of Veterinary Medicine, Kasetsart University, Bangkok 10900, Thailand

**Keywords:** equine melanocytic neoplasm, faecal proteome, lipid metabolism, functional analysis

## Abstract

Equine melanocytic neoplasm (EMN) is a common disease in older grey horses. The purpose of this study was to examine the potential proteins throughout EMN stages from faecal proteomic outlining using functional analysis. Faecal samples were collected from the rectum of 25 grey horses divided into three groups; normal group without EMN (*n* = 10), mild EMN (*n* = 6) and severe EMN (*n* = 9). Based on the results, 5910 annotated proteins out of 8509 total proteins were assessed from proteomic profiling. We observed differentially expressed proteins (DEPs) between the normal group and the EMN group, and 109 significant proteins were obtained, of which 28 and 81 were involved in metabolic and non-metabolic functions, respectively. We found 10 proteins that play a key role in lipid metabolism, affecting the tumour microenvironment and, consequently, melanoma progression. Interestingly, FOSL1 (FOS like 1, AP-1 transcription factor subunit) was considered as a potential highly expressed protein in a mild EMN group involved in melanocytes cell and related melanoma. Diacylglycerol kinase (DGKB), TGc domain-containing protein (Tgm2), structural maintenance of chromosomes 4 (SMC4) and mastermind-like transcriptional coactivator 2 (MAML2) were related to lipid metabolism, facilitating melanoma development in the severe-EMN group. In conclusion, these potential proteins can be used as candidate biomarkers for the monitoring of early EMN, the development of EMN, further prevention and treatment.

## 1. Introduction

Equine melanocytic neoplasm (EMN) is a kind of skin tumour that mainly affects older grey horses [1,2]. In contrast to humans, EMN does not seem to be induced by ultraviolet radiation exposure [3]. The first stage of EMN is localised only in dermal typical sites and classified as benign, with slow growth [4]. Subsequently, in an unpredictable period, the growth rate increases and metastases occur in internal organs, which was observed around 66% of horses suffering from EMN [5], contradicting the findings of MacGillivray et al. [4], who reported that 14% of dermal melanomas become malignant tumours and may form metastases in vital visceral organs.

The four melanocyte-related genes that mutate are nuclear receptor subfamily 4, group A, member 3 (NR4A3), syntaxin-17 (STX17), thioredoxin domain–containing 4 (TXNDC4) and inversin (INVS) [6]. However, the tumour develops from the higher regularity of the 4.6-kb duplication in intron 6 of the STX17 gene as a result of the defected vesicle transportation of melanosome cells to the hair follicle [6]. The confirmation of STX17 mutation is associated with gene NR4A3, with a higher presentation in EMN determined via the Northern blot and Real-Time PCR techniques [7]. In addition, the Agouti signalling protein (ASIP), which contributes to increased melanocortin-1 receptor pathway signalling, is responsible for increased susceptibility to melanoma [8].

Although EMN can easily be diagnosed because of its unique structural and positional characteristics, melanoma confirmation still requires fine needle aspiration cytology and/or biopsy for pathological examination, which is an invasive technique. In addition, there are limitations to the treatment, including the inability to prevent by using the cross-reaction of the HuTyr DNA vaccine [9], requiring further investigation. 

The advantages of proteomics can be used to analyse the modification state of proteins. Regarding equine melanoma, several studies have used antigen cell lines, but reports in vivo are scarce. Unlike in human medicine, proteomics plays an important role in predicting the responses to the treatment of cutaneous malignant melanoma (CMM) by chemotherapy of lymph node metastasis samples. Interestingly, there are four proteins expressed, namely cathepsin inhibitor cystatin B (CSTB), myo-inositol 1-phosphate synthase (ISYNA1), coagulation factor F13A1 and the calcium-binding protein (S100A13); S100A13 in CMM was overexpressed in non-responded or resistance to chemotherapy [10]. Moreover, proteomics can be a rapid, non-invasive tool in canine oral melanoma screening. A previous study used three different methods of saliva proteomics composed of matrix-assisted laser desorption/ionisation with time-of-flight mass spectrometry (MALDI-TOF MS) coupled with liquid chromatography-tandem MS (LC-MS/MS) [11], an in-gel digestion coupled with mass spectrometry (GeLC-MS/MS) [12] and in-solution digestion LC-MS/MS [13]. Although the results revealed different potential protein expressions, further investigations found that those putative proteins are linked in the same pathway. Notably, using biomarkers to screen or investigate EMN in a horse with a non-invasive technique could be the benefit to field practice. Recently, Tesena and colleagues [14] found potential proteins in a less invasive technique from serum EMN in grey horses, using the LC-MS/MS technique, which can be candidate protein biomarkers in mild and severe stages.

In this context, this study aimed to identify the differentially expressed proteins between normal grey horses and EMN grey horses using LC-MS/MS. We further performed functional analysis for early diagnosis of the EMN stage. In addition, functional analysis was performed for the identification of candidate biomarkers.

## 2. Materials and Methods

### 2.1. Ethics Information

The experiment was reviewed and approved by the Institution of Animal Care and Use Committee (IACUC) of the Faculty of Veterinary Science, Kasetsart University, Bangkok, Thailand, under permission No. ACKU63-VET-046; the owners of the horses provided an informed consent form.

### 2.2. Animals and Faecal Sample Collection

The grey horses were housed at the Horseshoe Point International Riding School, Chonburi, Thailand. In total, there were 25 grey horses, with Lusitano (*n* = 15), Thoroughbred (*n* = 3) and ponies (*n* = 7); the sexes included stallions (*n* = 2), geldings (*n* = 4) and mares (*n* = 19). The horses were aged between 10 and 31 years and weighed between 260 and 470 kg. Physical examination included vital sign checks (heart rate, respiratory rate, gut sound, mucous membrane and rectal temperature), and the positions of the lumps were recorded. Subsequently, one grey horse was subjected to surgery for confirmation of equine melanoma via histopathology. The horses were classified as EMN horses according to the system defined by Desser et al. [15]. After inspection of the melanomas, three veterinarians independently assessed the melanomas at the typical sites, including the underside of the tail, the perineum region along with the vulva or penis or scrotum, the perianal region, around the muzzle of the upper and lower lips and around the eyelids. The EMN category (normal, mild and severe EMN) was then modified by Tesena et al. (2021) [14], who reported serum proteomic profiling of EMN. Accordingly, the grey horses that were nodule-free were arranged as the normal group or grade 0. In addition, one solitary nodule with a diameter of 0.5 cm at typical location were arranged as the mild group or grade 1. Horses with melanomas with a diameter of 0.5–2 cm and/or one or more nodules with a diameter of 5 cm at typical locations, with severe progression including extensive confluent melanoma, exophytic tumors with wet surfaces and ulceration, and metastases in different organs, were classified as Grades 2–5 (severe), considered as invasive melanoma cases. Faeces samples were collected by rectal palpation, separated in a sterile microcentrifuge tube and kept in liquid nitrogen during transportation to the laboratory. Subsequently, the samples were kept at −80 °C until proteome analyses. Blood samples were collected from the jugular vein to obtain complete blood counts and serum blood chemistry profiles on creatinine, aspartate aminotransferase (AST), albumin and total protein [16]. The data on physical inspection and information on age, sex, breed and category of EMN are shown in Table 1.

Age, gender and breed were presented as arithmetic mean. Data on hematology and blood chemistry were analysed using one-way ANOVA with Duncan’s Multiple Range Test as post-hoc analysis, using groups as fixed factors. The statistically significant differences were considered at *p* < 0.05. All analyses were performed in RStudio 1.3.1056 [17] with the Rcmdr Package [18].

### 2.3. Faecal Sample Preparation

Approximately 0.1 g (100 mg) of faecal samples from each grey horse was dissolved in 0.1% SDS and centrifuged at 10,000× *g* for 10 min; the supernatant was kept in a new microcentrifuge tube. The total protein concentration was determined according to the Lowry method, using bovine serum albumin (BSA) as the standard [19]. Briefly, 5 µg of samples were subjected to in-solution digestion. Samples were completely dissolved in 10 mM ammonium bicarbonate (AMBIC), and the disulphide bonds were reduced using 5 mM dithiothreitol (DTT) in 10 mM AMBIC at 60 °C for 1 h, followed by alkylation of the sulfhydryl groups using 15 mM Iodoacetamide (IAA) in 10 mM AMBIC at room temperature for 60 min in the dark. Subsequently, the samples were mixed with 50 ng/µL of sequencing-grade trypsin (1:20 ratio) (Promega, Walldorf, Germany) and incubated at 37 °C overnight. The digested peptide samples were dried and dissolved with 0.1% formic acid before injection into the LC-MS/MS.

### 2.4. Liquid Chromatography-Tandem Mass Spectrometry (LC/MS-MS)

The tryptic peptide samples were injected into an Ultimate3000 Nano/Capillary LC System (Thermo Scientific, Waltham, Massachusetts, MA, USA) coupled to a HCTUltra LC-MS system (Bruker Daltonics Ltd.; Hamburg, Germany) equipped with a nano-captive spray ion source. Briefly, 5 µL of peptide digests were enriched on a µ-Precolumn 300 µm i.d. × 5 mm C18 Pepmap 100, 5 µm, 100 A (Thermo Scientific, Waltham, Massachusetts, MA, USA), separated on a 75 μm I.D. × 15 cm and packed with Acclaim PepMap RSLC C18, 2 μm, 100Å, nanoViper (Thermo Scientific, Waltham, Massachusetts, MA, USA). The C18 column was enclosed in a thermostated column oven set to 60 °C. Solvents A and B, containing 0.1% formic acid in water and 0.1% formic acid in 80% acetonitrile, respectively, were supplied to the analytical column. A gradient of 5–55% solvent B was used to elute the peptides at a constant flow rate of 0.30 μL/min for 30 min. Electrospray ionisation was carried out at 1.6 kV using the CaptiveSpray and nitrogen as drying gas (flow rate about 50 L/h). Collision-induced-dissociation (CID) product ion mass spectra were obtained using nitrogen gas as collision gas. Mass spectra (MS) and MS/MS spectra were obtained in the positive-ion mode at 2 Hz across the range (*m*/*z*) of 150–2200. The collision energy was adjusted to 10 eV as a function of the *m*/*z* value. The LC-MS analysis of each sample was performed in triplicate.

### 2.5. Quantification and Identification of Proteins

We used the DeCyder MS Differential Analysis software (DeCyderMS, GE Healthcare, Uppsala, Sweden) to quantify the proteins in individual samples; the Mascot search engine was applied to correlate the MS/MS spectra to the Uniprot mammal database [20,21], which was accessed on 23 February 2021. The Mascot’s standard settings were as follows: maximum of three missed cleavages, mass tolerance of 0.6 Dalton for the main search, trypsin as digesting enzyme, carbamidomethylation of cysteine as fixed modification, oxidation of methionine as variable modifications and peptide charge state (1+, 2+ and 3+). Visualisation and statistical analyses were conducted using the MultiExperiment Viewer (MeV) in the TM4 suite software [22]. Proteins considered as identified proteins had at least one peptide with an individual mascot score corresponding to *p* < 0.05. The maximum peptide intensities level in log_2_-transformed to Microsoft Excel was then established in a normal grey horse group, mild EMN group and severe EMN group. As a result, raw MS/MS spectra data were examined as protein sequences and PELs of the normal, mild EMN and severe EMN stages. Subsequently, a Venn diagram was generated to show the differences among proteins originating from differential analyses [23]. The raw MS/MS spectra data are available in ProteomeXchange: JPST001422 and PXD030474.

### 2.6. Functional Analysis

**Figure 1:**
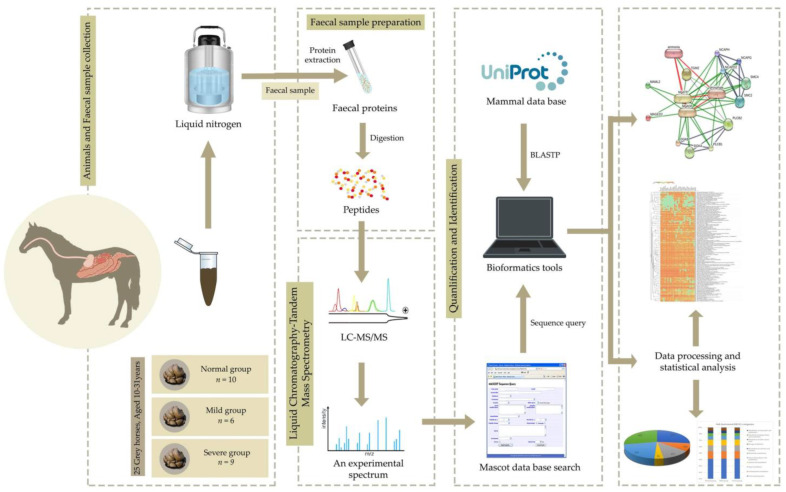
The proteomic framework from EMN faecal sample towards protein identification and quantification.

Differentially expressed protein (DEP) analysis was conducted as previously described [14]. Specifically, identification of the potential proteins throughout EMN stages and analysis of differentially expressed proteins (DEPs) were performed for normal, mild and severe EMN stages. The Wilcoxon rank-sum test and multiple testing via false discovery rate (FDR) correction were used to identify a significant protein among the groups (adjusted *p*-value < 0.05). The significant protein expression was intended to serve as a potential protein to classify normal and severe EMN in horses. For the functional annotation of DEPs, the KEGG database [24] was used. The framework of proteomic characterization contributing to DEP analysis and functional annotation is shown in Figure 1.

## 3. Results

The haematology and serum blood chemistry of horses in different group are shown in Table 2. Most blood parameters did not differ among the studied groups and were in the reference ranges (*p*-value > 0.05). Lower platelets numbers were observed in the mild EMN group compared to the normal group (*p*-value < 0.05); however, all values were still within the reference range.

### 3.1. The Functional Annotation Analysis Results 

All 5910 annotated proteins out of 8509 total proteins could be partitioned into six main functional categories according to the KEGG database, namely environmental information processing, metabolism, genetic information processing, cellular processes, human diseases and organismal systems. The proteins with functional annotation analysis results for the grey horses without EMN and the EMN groups with early and severe stages are summarised in Table 3. Although the number of proteins in each group differed, the percentages for all three groups were similar. The percentages of the main functional proteins were as follows: environmental information processing (28%), metabolism (26%), genetic information processing (21%), cellular processes (12%), human diseases (9%) and organismal systems (4%).

Remarkably, the greatest numbers of proteins involved in metabolic functions were related to carbohydrate metabolism, followed by those related to lipid metabolism, amino acid metabolism, glycan biosynthesis and metabolism, nucleotide metabolism, metabolism of cofactors and vitamins and energy metabolism. The other sub–functional categories are listed in Table 4.

### 3.2. Analysis of Differentially Expressed Proteins and Functional Annotation of Grey Horses across EMN Stages 

**Figure 2:**
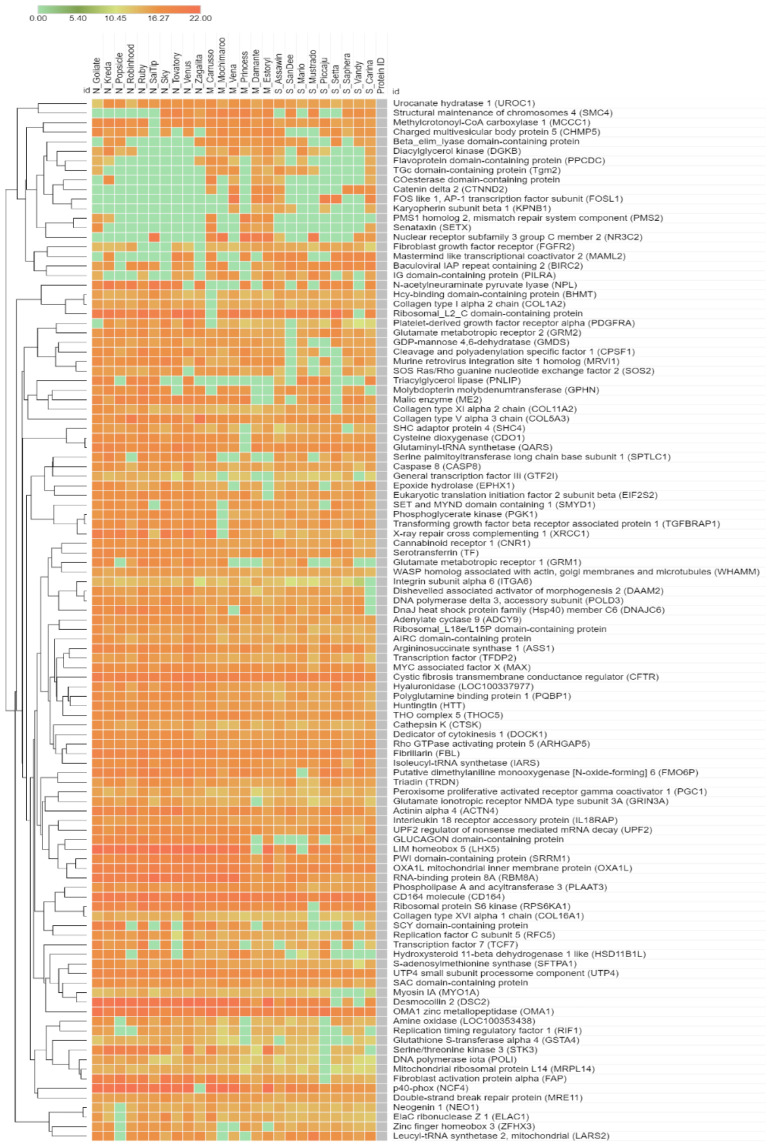
The comparative protein expression levels across a normal grey horse group, mild EMN group and severe EMN group. The heatmap is generated from the intensity of significant proteins database for each group using the CLUE program (https://clue.io/command, accessed on 10 September 2021). N, M and S represent normal, mild and severe, respectively.

A total of 5910 annotated proteins from the assessed proteomic data were included in the DEP analysis with the Wilcoxon rank-sum test and FDR correction. Regarding the list of DEPs, 109 significant proteins were identified under an adjusted *p*-value < 0.05. Among these proteins, 109 significant proteins were categorised as functional protein according to the KEGG database as listed in Table S1. Among these, we found 28 proteins involved in metabolism and 81 proteins involved in non-metabolism, i.e., cellular processes (15 proteins), environmental information processing (28 proteins), genetic information processing (26 proteins), organismal systems (5 proteins) and human diseases (7 proteins). Figure 2 illustrates all groups based on the significant results for comparative protein expression levels (PELs).

Using the 109 significant proteins, potential proteins were selected from faecal samples using notwithstanding the median values in the mild and severe groups, while no protein expression occurred (level 0) (Table 5). In addition, we also display the potential proteins from serum samples from a previous study to compare the associated functional proteins [14].

As shown in Table 5, we observed proteins involved in both metabolic and non–metabolic functions. According to the significant results for comparative protein expression levels (PELs), they were classified into two categories: (1) potential proteins expressed only in mild-stage EMN and (2) potential proteins highly expressed in mild and severe stages of EMN. In the faecal proteome, four potential proteins were involved in metabolism, including diacylglycerol kinase (DGKB), flavoprotein domain–containing protein (PPCDC), beta_elim_lyase domain–containing protein and COesterase domain–containing protein, whereas the other 10 potential proteins were involved in non–metabolic functions.

**Figure 3:**
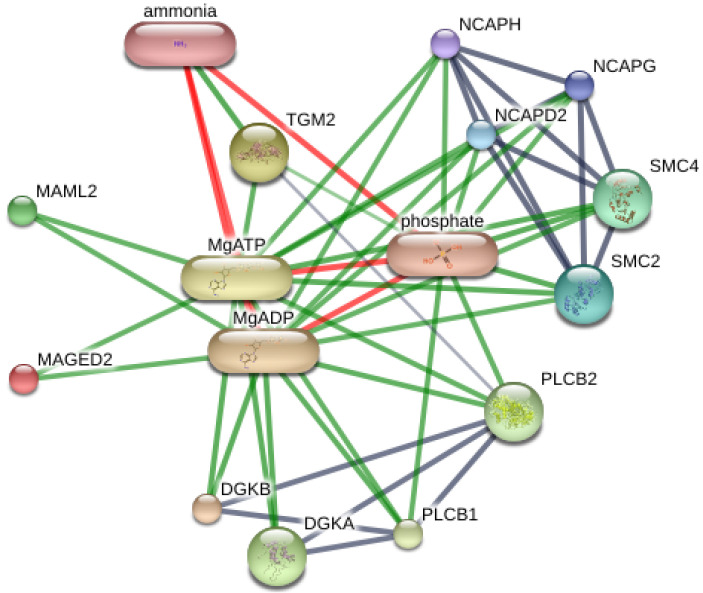
Contribution of DGKB, MAML2, SMC4, TGM2 and MAGED2 in networks of protein-protein interaction and protein-chemotherapy interaction of Equus caballus. Abbreviations: DGKA; diacylglycerol kinase alpha, DGKB; diacylglycerol kinase beta, MAML2; mastermind like transcriptional coactivator 2, SMC4; structural maintenance of chromosomes 4, TGM2; transglutaminase 2, MAGED2; melanoma antigen family D2 in Equus caballus that computational prediction between organism and interaction aggregated by the Stitch program (www.stitch.embl.de, accessed on 10 September 2021) Version 5.0.

Using 14 significant faecal proteins, we selected all significant results to predict interactions with chemicals and organism-related melanoma from computational prediction and interaction, aggregated by the STITCH Version 5.0 (www.stitch.embl.de, accessed on 10 September 2021). As shown in Figure 3, this feature provided the comprehensive protein expression pattern with four significant proteins, including diacylglycerol kinase (DGKB), mastermind-like transcriptional coactivator 2 (MAML2), structural maintenance of chromosomes 4 (SMC4) and TGc domain-containing protein (Tgm2). As 10 significant proteins were not present, further studies are necessary to discover melanoma-related information.

## 4. Discussion

Equine melanocytic neoplasm (EMN) is the most common malignant tumour in older grey horses [2]. It normally is benign at the early stage, and two-thirds of the affected horses develop cancer in an advanced stage [4]. Disease prognosis depends on the stage of the cancer at diagnosis [25]. Early diagnosis increases the chances of survival. Histological investigation is the gold standard to diagnose the cancer [26,27]. However, the procedure is invasive. Recently, cancer screening by non-invasive faecal biomarkers has been described [28]. In this study, we identified a number of differentially expressed faecal proteins by mass spectrometry for the normal group and different stages of EMN as faecal biomarkers for EMN diagnosis, prognosis and disease monitoring. There were some limitations in this research. Because the horse owner refused to enable researchers to collect the sample, the grey horses were graded as mild group or grade 1 based on inspection and palpation in specified locations without further diagnostic techniques. Following the previous recommendations, three veterinarians independently assessed the melanomas to confirm the diagnosis of melanoma. As a result, it may be sufficient to ensure this classification for this group.

Four proteins identified in the faecal proteome are potential biomarkers of the cancer, including diacylglycerol kinase (DGKB, DGK), mastermind-like transcriptional coactivator 2 (MAML2), structural maintenance of chromosomes 4 (SMC4), IG domain-containing protein and transglutaminase 2 (Tgm2). These proteins were highly expressed in EMN groups of mild and severe stages but cannot found or low intensity in normal group. Diacylglycerol kinase (DGKB, DGK) is involved in metabolism, whereas mastermind-like transcriptional coactivator 2 (MAML2), structural maintenance of chromosomes 4 (SMC4), IG domain-containing protein and transglutaminase 2 (Tgm2) are in the non-metabolism category.

The DGK represents a family of lipid kinases that convert diacylglycerol (DAG) to phosphatidic acid (PA), using ATP as a phosphate source [29]. The DAGs serve as basic components of membranes [30] and lipid signalling molecules for protein kinase stimulation; they are signalling molecules that regulate distinct signalling pathways for a variety of cellular processes [31], especially the lipid metabolic pathway [32]. Many studies have reported that DGK plays a role in cancer progression, invasion, migration and support the survival of lymphoma, hepatoma and melanoma [33]. Moreover, DGK encourages the nonresponsive state of T and NK cells in the tumour microenvironment [34], which can play an important role in cancer elimination, resulting in cancer therapeutic resistance [35].

Recently, we reported anaplastic lymphoma kinase (ALK), a potential serum protein related to EMN [36]. It is a transmembrane tyrosine kinase receptor related to melanoma by promoting the metabolism signalling cascade pathway. The DGKs regulate signalling by anaplastic lymphoma kinase (ALK). Both faecal DGK and serum ALK levels are related to cancer cell growth promotion. Interestingly, both DGK and ALK could not be found in the normal group but were highly expressed in mild and severe EMN stages, as shown in Table 5.

Accordingly, Tgm2 is an enzyme of the transglutaminase (TG) family that can catalyse the post-translational modification of proteins. In addition, Tgm2 mediates the cross-link of extracellular matrix (ECM) proteins, can hydrolyse nucleotide guanine triphosphate (GTP) and serves as a protein for maintaining ECM stability. In addition, it has multiple functions including regulation of cellular proliferation, as a receptor-mediated endocytosis, and stimulates the cellular adhesive capacity of fibroblasts [36]. Hence, Tgm2 is related to inflammation and modulates the roles in various tumours [37], including melanoma [38]. Moreover, Tgm2 regulates various biological comportments of tumour cells, including differentiation, invasion, adhesion, apoptosis, migration, proliferation, survival, growth, interaction with microenvironment metastasis, angiogenesis and chemoresistance through mechanisms including pro-crosslinking, protein binding, signal transduction and transformation via microvesicles [39]. This study reveals that the expression levels of Tgm2 in early and severe stages were up to 13–15-fold (median value) and 7–11-fold (average value) higher than those in the normal group (File S1). In humans, Tgm2 expression in melanoma samples were up to 24-fold during progressive stages [39].

The MAML2 acts as a transcription coactivator and is associated with the Notch signalling pathway, the positive regulation of transcription by RNA polymerase II and capable of developing a multiprotein complex [40]. It plays a role in disturbing the normal cell cycle and in the differentiation and development of the tumour [41]. Regarding melanoma, the Notch signalling pathway is related with melanoblasts, including maintenance of the undeveloped stage, appropriate location controlling and prevention of differentiated melanocytes [42]; therefore, the Notch signalling pathway can be activated in skin melanoma [43]. It is upregulated in various tumours including glioblastoma multiforme (GBM) the brain tumous [44], mucoepidermoid carcinoma [45] and breast cancer [46].

The SMC4 is a protein complex in the family of chromosomal ATPases and plays a role in regulating chromosome assembly and seclusion [44]. It is upregulated in various malignancies, including lung cancer [45], breast cancer [46], liver cancer [47] and, in particular, colorectal cancer, with a high SMC4 expression when compared with normal tissue [44]. It is also associated with tumour proliferation and tumour cell development [48]. So far, there are no studies on SMC4 and equine melanoma; however, it may affect chromosomal stability via the p53 pathway in breast cancer [49], and chromosomal rearrangements from increased double-strand DNA disruptions may lead to mutation and mismatches [46].

### 4.1. Identification of Differently Expressed Proteins in Mild-Stage EMN

Several proteins were expressed differently in the mild stage, such as flavoprotein domain-containing protein, FOS-like 1; AP-1 transcription factor subunit (FOSL1), catenin delta 2 (CTNND2), karyopherin subunit beta 1 (KPNB1), PMS1 homolog 2, mismatch repair system component (PMS2) and senataxin (SETX).

Flavoproteins contain a nucleic acid derivative of riboflavin and play an important role in mitochondrial electron transport, fatty acid degradation and redox regulation [50]. Regarding molecular functions, they have catalytic activity, including the removal of radicals contributing to oxidative stress, photosynthesis and DNA repair [51]. Therefore, the uncharacteristic expression of flavoproteins could lead to degenerative changes in melanoma or in the nervous system and peripheral neuropathy, affecting the proliferation and mobility of various cancer cells such as breast cancer, gastric, colorectal and lung cancer cells [52].

Surprisingly, FOSL1 is one of several proteins in the AP-1 transcription factor family and considered as a regulator of cell proliferation, differentiation and transformation. In addition, it is the main immediate early AP-1 participant encouraged by melanoma. In a previous study, FOSL1 was highly expressed in melanocytes, facilitating tumour growth [53]. Teutschbein and colleagues [54] discovered that FOSL1 had increased expression levels in human melanoma cell lines when compared to human melanocytes, whereas FOSL1 reduction in human melanoma cell lines also reduced migration and proliferation. 

The CTNND2 plays a crucial role in biological processes such as neuronal development, particularly in the formation and/or maintenance of dendritic spines and synapses; it is also involved in the regulation of Wnt signalling as amplified in oesophageal carcinoma [55]. These functions in Wnt signalling regulate gene expression and modulate Rho GTPases of the Ras superfamily in cytoskeletal reorganisation, as observed by Tesena [14] and colleagues, who reported that a Rho-binding domain leads to multiple cell modifications in equine melanoma that interact with lipid modification. It also probably acts on beta-catenin turnover in neurons [56], and prostate cancer can be a result of overexpression of beta-catenin [57]. In addition, it is involved in neuronal cell adhesion and tissue morphogenesis and integrity by regulating adhesion molecules [57].

The PMS2 is an enzyme involved in mismatch repair as well as latent endonuclease action, introducing marks into a discontinuous DNA component [58]. Diseases associated with PMS2 mutation include colorectal cancer, hereditary nonpolyposis, type 4 and Mismatch Repair Cancer Syndrome. The mutations in the promoter region of PMS2 are significantly associated with high tumour mutational burden (TMB), especially melanoma [59].

The SETX is the protein involved in lysosomal degradation and autophagy. It has helicase activity, acts in identical protein binding and transcription termination site sequence-specific DNA binding [60]. In addition, SETX is supposed to be expressed in the mild stage through the formation of autophagosomes [61]. Diseases involving SETX mutations are usually associated with the nervous system, including ataxia with oculomotor apraxia type 2 (AOA2) [62] and a juvenile form of amyotrophic lateral sclerosis named ALS4 [63].

The KPNB1 is the nuclear transport receptor that is responsive to the importins and exportins, the pores located between nucleus and cytoplasm. Importins and exportins are dependent on the specific nuclear localisation (NLS) and the nuclear export signal, respectively [64]. The KPNB1 plays crucial roles in mitotic and chromosomal integrity, influencing the biology of the cancer cell [65]. Dysregulation of the expression of the nuclear transporter has been used as the tool for the prognosis of breast cancer, brain cancer, gastric cancer, prostate cancer, ovarian cancer, bladder cancer, liver cancer, lung cancer, oesophageal cancer and melanoma [66]. We therefore assume that proteins of mild–stage EMN were expressed but did not lead to metastasis.

### 4.2. Proteins Involved in Lipid Metabolism and Linked to EMN

Following the evaluation of proteomics data by the KEGG database, 5910 annotated proteins from the faecal proteome were analysed using the Wilcoxon rank-sum test and FDR correction with an adjusted *p*-value of 0.05. Additionally, the list of DEPs, 10/28 significant faecal proteome and 7/10 significant serum proteome revealed the proteins of interest involved in lipid metabolism (Table S2). 

This study discovered that the expression protein involved in lipid metabolism appeared with a higher percentage than those involved in other functions in the main metabolism category of both the serum and faecal proteomes. Furthermore, fatty acid synthesis and oxidation play an important role in the development of EMN [67] by improving ATP in tumour cells, especially during metabolic stress responses [68]. Fatty acid biosynthesis can be maintained during the rapid spread of EMN processes or when tumour cells are starved of nutrients [69].

Lipid metabolism is composed of two major pathways: fatty acid biosynthesis (anabolism) and fatty acid breakdown into acetyl CoA via beta-oxidation (catabolism) [70]. There are several pathways to synthesise lipids from the lipid membrane, triacylglycerols and cholesterol. Membrane lipid biosynthesis produces two types of membrane lipids: sphingolipids and glycerophopholipids [71]. The precursor of triglyceride biosynthesis is phosphatidic acid, and phosphatidylinositol acid phosphorylation translates this to diacylglyceride and then to triacylglyceride by acyltransferase [72]. Another example is fatty acid biosynthesis, where acetyl-CoAserves as a precursor to malonyl-CoA, which is then assembled to form palmitate and palmitic acid. The isoprenoid pathway converts acetyl-CoA, which is transformed into acetoacetyl-CoA and then converted into cholesterol. Furthermore, before entering the cell, triglyceride is decomposed into fatty acid and glycerol via lipid catabolism. In the glycolysis pathway, glycerol is converted to glyceraldehyde-3-phosphate, which is then oxidised to generate energy. Following that, long-chain fatty acids in the acyl-CoAformat will cross the mitochondrial membrane. Fatty acid catabolism begins in the cytoplasm as acyl-CoA and then moves to the mitochondrial membrane to begin the process of beta oxidation. The primary products of the beta oxidation pathway are acetyl-CoA, NADH and FADH [73].

As previously stated, EMN develops from neural crest-derived melanocytes that are located at the dermal-epidermal junction of the skin. The first stage of EMN growth, beginning with the tumour cell, is the interaction with keratinocytes in the surrounding microenvironment [74]. Following that, EMN progresses by invading mostly populated adipocytes through the subcutaneous layer. Melanoma cells make direct contact with adipocyte tissue and spread to other organs in both the local and metastasis tumour microenvironments. Furthermore, the tumour cell microenvironment releases lipids, which can result in EMN growth, proliferation and invasion [75].

In this study, we discovered that the majority of proteins involved in lipid metabolism are expressed in both serum and faecal samples, which is consistent with other studies on lipid metabolic reprogramming in melanoma. These findings revealed an increasing progression of the tumour microenvironment surrounding it as well as the role of fatty acid metabolism in melanoma cell aggressiveness. Moreover, the expression of these proteins is linked to significant lipogenic synthesis, which is associated with tumour cell invasion and poor prognosis. In addition, alterations in the lipid metabolic network can sustain cell growth and metastasis in melanoma cells [76]. 

The EMN is heavily involved in lipid metabolism, which is typically measured using triglyceride and cholesterol levels as indicators of lipid and energy metabolism. In equines, this is most commonly characterised by increased triglyceride concentrations, with variable increases in cholesterol as the balance between fat mobilisation and use becomes dysregulated [77]. Triglyceride levels in the blood reflect both dietary intake and hepatic synthesis. There are several fatty acid sources that can be used to synthesise triglycerides [78]. Furthermore, the cause of lipid metabolism disorder, which is characterised by abnormal triglyceride and cholesterol levels, could be hereditary or acquired, as well as a systemic inflammatory response syndrome [79].

## 5. Conclusions

In this study, using proteomics profiles and bioinformatic methods, we identified and related biomarkers for EMN that have previously been identified in other studies. This study used non-invasive approaches to identify the novel biomarker from faeces in grey horses. The protein FOSL1 was only expressed in mild stages. Furthermore, the high levels of DGKA, MAML2, SMC4 and Tgm2 found in this study make these proteins potential biomarkers for EMN. Proteins linked to lipid, such as DGKB, could be used as additional biomarkers for non-invasive EMN. As a result, in EMN grey horses, the LC-MS/MS technique and an integrated protein platform can be used for early diagnosis, improving therapy efficacy.

## Figures and Tables

**Table 1:** Signalments, clinical classification and EMN category of grey horses.

ID	Age (Years)	Sex	Breed	The Location of Tumor	EMN Categories *
Normal 1	12	Mare	Lusitano	-	0
Normal 2	13	Mare	Lusitano	-	0
Normal 3	13	Mare	Lusitano	-	0
Normal 4	15	Mare	Pony	-	0
Normal 5	15	Mare	Lusitano	-	0
Normal 6	15	Mare	Thoroughbred	-	0
Normal 7	18	Mare	Lusitano	-	0
Normal 8	19	Mare	Lusitano	-	0
Normal 9	19	Mare	Lusitano	-	0
Normal10	26	Gelding	Pony	-	0
Mild 1	15	Gelding	Lusitano	Underside of the tail	1
Mild 2	18	Mare	Lusitano	Underside of the tail	1
Mild 3	18	Mare	Lusitano	Underside of the tail	1
Mild 4	22	Mare	Pony	Underside of the tail	1
Mild 5	24	Mare	Lusitano	Underside of the tail	1
Mild 6	31	Mare	Pony	Underside of the tail	1
Severe 1	10	Gelding	Thoroughbred	Underside of the tail, peri-anal	2
Severe 2	17	Gelding	Lusitano	Underside of the tail, peri-anal	2
Severe 3	26	Stallion	Pony	Underside of the tail, peri-anal	2
Severe 4	29	Stallion	Lusitano	Underside of the tail, peri-anal	2
Severe 5	22	Mare	Pony	Underside of the tail, peri-anal, vulva	3
Severe 6	23	Mare	Lusitano	Underside of the tail, peri-anal, vulva	4
Severe 7	23	Mare	Thoroughbred	Underside of the tail, peri-anal, vulva	2
Severe 8	26	Mare	Pony	Underside of the tail, peri-anal, vulva	2
Severe 9	26	Mare	Lusitano	Underside of the tail, peri-anal, vulva	3

* EMN categories: The nodule-free grey horses served as the normal group or grade 0. In addition, one solitary nodule with a diameter of 0.5 cm at a typical location were arranged as the mild group or grade 1. Horses with melanomas with a diameter of 0.5–2 cm and/or one or more nodules with a diameter of 5 cm at typical locations, with severe progression including extensive confluent melanoma, exophytic tumors with wet surfaces and ulceration, and metastases in different organs, were classified as Grades 2–5 (severe).

**Table 2:** The haematology and serum blood chemistry profiles of horses with different EMN stages.

Parameters		Groups			SEM	*p*-Value	Reference Range
		Normal	Mild EMN	Severe EMN			
**Haematology**							
	WBC (cell/mm^3^)	10.7	9.5	11.5	0.469	0.30	5.6–12.10
	Neutrophils	5.43	5.34	6.82	0.311	0.08	5.2–7.0
	Band neutrophils (Cell/mm^3^)	0.00	0.00	0.00	–	–	0–1
	Lymphocyte	4.85	3.83	4.14	0.381	0.56	2.1–4.2
	Monocyte	0.36	0.25	0.34	0.020	0.08	0–6
	Eosinophils	0.09	0.10	0.15	0.031	0.70	0–7
	Basophils	0.00	0.00	0.00	–	–	0–0.2
	Platelets	249 ^b^	196 ^a^	230 ^a,b^	8.228	0.04	117–256
	RBC (cell/mm^3^)	7.55	7.68	7.47	0.176	0.90	6.0–10.4
	Hematocrit (%)	33.7	34.2	34.4	0.800	0.94	27–43
	Hemoglobin	11.1	11.4	11.3	0.255	0.91	10.1–16.1
	MCV	44.8	44.9	46.1	0.480	0.48	37–49
	MCH	14.8	14.9	15.1	0.129	0.54	13.7–18.2
	MCHC	33.1	33.5	32.9	0.211	0.62	35.3–39.3
	RDW–CV	26.6	26.2	26.6	0.507	0.95	–
	Fibrinogen	420	633	567	63.32	0.39	100–400
**Blood chemistry**							
	Creatinine	1.82	1.67	1.87	0.044	0.22	0.4–2.2
	Total protein	7.05	6.65	6.96	0.081	0.15	5.6–7.6
	Albumin	3.34	3.30	3.32	0.027	0.85	2.6–4.1
	AST (SGOT)	280	289	284	8.533	0.91	160–412

^a b^ Means in the same row with different superscripts were different (*p* < 0.05) (One–way ANOVA). SEM is standard error of the mean.

**Table 3:** Main functional KEGG categories 3 groups of the experimental grey horses EMN of the faeces sample.

Main–Functional KEGG Categories	Groups		
	Normal Grey Horse	Mild EMN	Severe EMN
Metabolism	573	572	573
Human diseases	189	189	189
Cellular processes	255	255	257
Organismal systems	81	82	82
Genetic information processing	450	450	454
Environmental information processing	602	612	614

**Table 4:** Sub–functional KEGG categories 3 groups of the experimental grey horses EMN of the faeces sample.

Sub–Functional KEGG Categories	Groups		
	Normal Grey Horse	Mild EMN	Severe EMN
Lipid metabolism	107	106	107
Energy metabolism	38	38	38
Nucleotide metabolism	52	52	52
Amino acid metabolism	104	105	105
Carbohydrate metabolism	139	138	139
Metabolism of other amino acids	9	9	9
Glycan biosynthesis and metabolism	66	66	66
Metabolism of cofactors and vitamins	48	48	47
Metabolism of terpenoids and polyketides	4	4	4
Xenobiotics biodegradation and metabolism	6	6	6

**Table 5:** Comparative significantly potential proteins of faecal proteome and serum proteome.

Protein ID	Protein Function	PELs ^a^		
		N	M	S
This study; significantly expressed faecal proteins				
A0A5F9CKF0	Diacylglycerol kinase (DGKB)	0 (6/10)	14.9254	12.529
H0VXN6	TGc domain-containing protein (Tgm2)	0 (9/10)	15.3301	13.542
A0A5F9C4M4	IG domain-containing protein (PILRA)	0 (6/10)	15.2587	14.44
G1U159	Beta_elim_lyase domain-containing protein	0 (7/10)	16.1641	15.13
A0A5F9CRH3	Structural maintenance of chromosomes 4 (SMC4)	0 (6/10)	17.5259	15.934
H0VCJ5	Mastermind like transcriptional coactivator 2 (MAML2)	0 (7/10)	8.15043	17.374
A0A286Y4H6	Nuclear receptor subfamily 3 group C member 2 (NR3C2)	0 (9/10)	19.3616	0 (5/9)
G1PHB6	FOS like 1, AP-1 transcription factor subunit (FOSL1)	0 (10/10)	8.73988	0 (5/9)
H0VZS6	Catenin delta 2 (CTNND2)	0 (10/10)	15.8651	0 (5/9)
G1PQ83	Karyopherin subunit beta 1 (KPNB1)	0 (10/10)	6.99422	0 (5/9)
A0A5F9C6H5	Flavoprotein domain-containing protein (PPCDC)	0 (7/10)	14.9349	0 (6/9)
A0A286XHI2	COesterase domain-containing protein	0 (9/10)	14.5562	0 (7/9)
H0UWB8	Senataxin (SETX)	0 (9/10)	14.9092	0 (8/9)
H0UU64	PMS1 homolog 2, mismatch repair system component (PMS2)	0 (8/10)	16.1875	0 (9/9)
Previous study; significantly expressed serum proteins				
Q9BDT7	BRCA1 (Fragment)	16.73	17.77	17.90
G1U3S4	Phosphorylase b kinase regulatory subunit (PHKA1)	16.30	18.29	18.33
G1SEQ3	Tyrosine-protein kinase receptor (ALK)	0 (7/10)	20.00	19.52
H0VGZ3	Rho-associated protein kinase (ROCK1)	0 (6/10)	18.55	17.69
PLPP6	phospholipid phosphatase 6	0 (10/10)	13.57	0 (7/10)
G1PKF6	sodium/potassium-transporting ATPase subunit alpha	0 (10/10)	14.68	14.22

N, M, S represent normal, mild and severe stages of EMN, respectively. ^a^ Median value of PELs is presented.

## Data Availability

Raw MS/MS spectra data are available in ProteomeXchange: JPST001422 and PXD030474.

## Supplementary Materials

The following are available online at https://www.mdpi.com/article/10.3390/vetsci9020094/s1. Table S1: List of 109 candidate proteins from differentially expressed proteins analysis under Wilcoxon rank–sum test and multiple testing via false discovery rate (FDR) correction between normal, mild and severe EMN stages. Table S2: The uniquely proteins involved in lipid metabolism of faecal and serum proteome.
